# Mapping Surface Potential in DNA Aptamer–Neurochemical and Membrane–Ion Interactions on the SOS Substrate Using Terahertz Microscopy

**DOI:** 10.3390/bios15010046

**Published:** 2025-01-13

**Authors:** Kosei Morita, Yuta Mitsuda, Sota Yoshida, Toshihiko Kiwa, Jin Wang

**Affiliations:** Graduate School of Interdisciplinary Science and Engineering in Health Systems, Okayama University, Okayama 700-8530, Japan; ps0o4miw@s.okayama-u.ac.jp (K.M.); p07k4tj7@s.okayama-u.ac.jp (Y.M.); pz9f0009@s.okayama-u.ac.jp (S.Y.); kiwa@okayama-u.ac.jp (T.K.)

**Keywords:** terahertz chemical microscope, surface potential, DNA aptamer–neurochemical complexes, membrane–ion interactions, SOS substrate, artificial cerebrospinal fluid

## Abstract

In this study, we utilized a terahertz chemical microscope (TCM) to map surface potential changes induced by molecular interactions on silicon-on-sapphire (SOS) substrates. By functionalizing the SOS substrate with DNA aptamers and an ion-selective membrane, we successfully detected and visualized aptamer–neurochemical complexes through the terahertz amplitude. Additionally, comparative studies of DNA aptamers in PBS buffer and artificial cerebrospinal fluid (aCSF) were performed by computational structure modeling and terahertz measurements. Beyond neurochemicals, we also investigated calcium ions, measuring their concentrations in PDMS-fabricated micro-wells using minimal sample volumes. Our results highlight the capability of TCM as a powerful, label-free, and sensitive platform for the probing and mapping of surface potential arising from molecular interactions, with broad implications for biomedical diagnostics and research.

## 1. Introduction

The measurement of surface potential plays a critical role in a diverse range of applications spanning chemical processes, engineering, biological research, and industrial production. Applications include advancements in sensing technologies, catalysis, corrosion prevention, cell adhesion studies, nanoparticle separation, pulp processing, and so on [1,2,3,4,5,6,7,8,9,10,11,12]. A recent study suggests that surface potential is a fundamental parameter for deciphering and interpreting complex electrical activities within biological systems. It provides essential insights into electrophysiological processes, enabling researchers and practitioners to explore dynamic interactions at the molecular and cellular levels [13].

Surface potential sensing is a powerful method for investigating molecular interactions. Small changes in surface potential can indicate binding events, such as neurotransmitters interacting with aptamers or ions interacting with selective membranes. DNA aptamers, known for their high specificity and stability, are particularly effective for targeting neurotransmitters like serotonin and dopamine. When anchored on electrochemical sensor or optical sensor surfaces, these aptamers create detectable changes in the local charge environment, allowing for label-free and highly sensitive detection of binding events [14,15,16,17]. The ability to detect and map subtle changes in surface potential is vital for advancing our understanding of molecular recognition processes on solid substrates. When target molecules—such as neurotransmitters or ions—bind to the surface, they alter the local charge environment, revealing key information about their presence and behavior. DNA aptamers play a critical role in this process, providing a robust, label-free platform for accurately monitoring these interactions.

Many advanced technologies have been widely developed for surface potential detection, each offering unique advantages in specific applications. These include Kelvin probe microscopy (KVM) [4], which enables non-contact measurement of surface potential with high spatial resolution, and field-effect transistors (FETs) [18,19,20], which provide highly sensitive and real-time monitoring of surface charge changes. Light-addressable potentiometric sensors (LAPS) facilitate spatially resolved measurements of potential changes using optical excitation [21,22,23,24,25], while electrochemical devices offer robust platforms for detecting surface charge dynamics through redox reactions and ionic conductivity [26].

Unlike those technologies, a terahertz chemical microscope (TCM) provides a label-free and highly sensitive approach for mapping surface potential by measuring terahertz amplitude [27,28,29,30,31]. TCM utilizes a silicon-on-sapphire (SOS) substrate and femtosecond laser pulses to generate terahertz waves, whose intensity is modulated by the underlying surface potential. This modulation enables the detection and quantification of neurotransmitters and ions by correlating their presence and concentration with changes in terahertz amplitude.

## 2. Materials and Methods

### 2.1. Reagents and Materials

Reagents including acetone (99.5%), ethanol (99.5%), sodium hydroxide (NaOH, 200 mM in Milli-Q water), and 3-aminopropyltriethoxysilane (APTES) were purchased from Sigma-Aldrich (Saint Louis, MO, USA). Bis(sulfosuccinimidyl)suberate (BS3) and phosphate-buffered saline (PBS) were obtained from Thermo Fisher Scientific (Waltham, MA, USA) and prepared at 10 mM in PBS as cross-linkers. Serotonin- and dopamine-binding aptamers were synthesized by Fasmac Co., Ltd, Tokyo, Japan. The aptamer concentrations (1800 ppm) were prepared in PBS or artificial cerebrospinal fluid (aCSF). Surface-blocking reagent ethanolamine-HCl was purchased from GE Healthcare (Chicago, IL, USA) and diluted to 0.1 M in Milli-Q water for use. Serotonin and dopamine solutions were prepared in PBS or aCSF at various concentrations (100 ppt to 1000 ppb for serotonin). For the ion measurements, a calcium ion-selective membrane solution was prepared from polyvinyl chloride (PVC), bis(2-ethylhexyl) sebacate (DOS), sodium ionophore II, and sodium tetrakis[3,5-bis(trifluoromethyl)phenyl]borate, dissolved in tetrahydrofuran (THF). Polyvinyl chloride was purchased from Wako Pure Chemical Industries, Ltd, Tokyo, Japan. DOS and sodium ionophore II were purchased from Sigma-Aldrich. PDMS (SYLGARD184) was purchased from Sansho Co., Ltd, Osaka, Japan.

### 2.2. SOS Substrate and Terahertz Chemical Microscope Optical Setup

**Figure 1:**
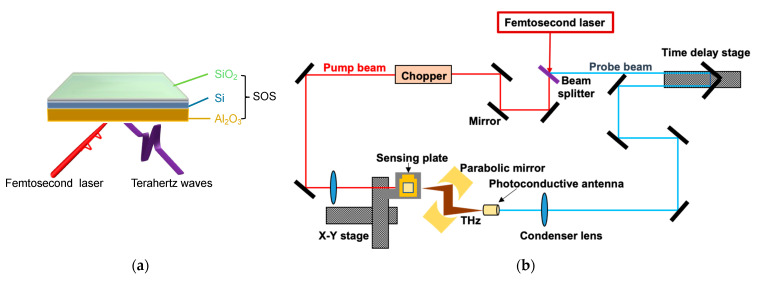
Schematic diagram of SOS substrate (**a**) and TCM optical setup (**b**).

**Figure 2:**
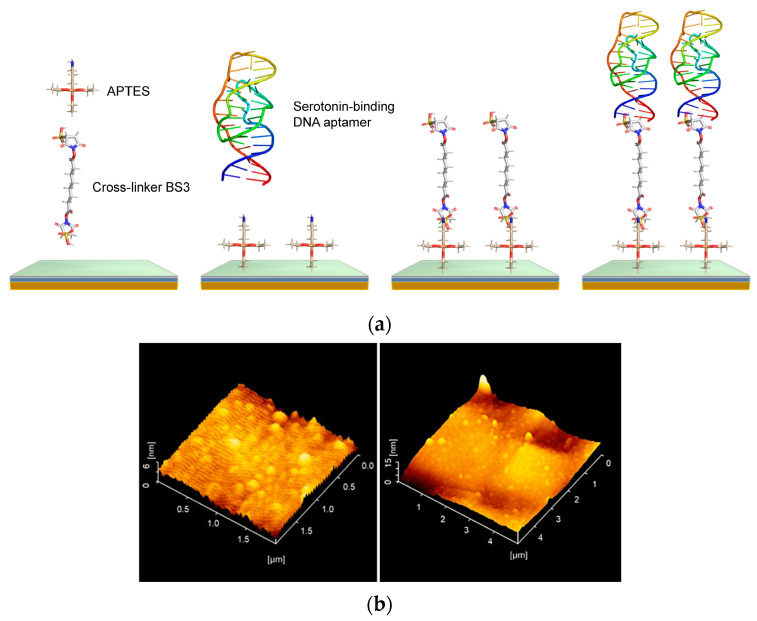
(**a**) Surface functionalization on SOS substrate with serotonin-binding DNA aptamers; (**b**) AFM image of serotonin-binding DNA aptamers.

The TCM is an instrument designed to extract information on chemical reaction events occurring on a terahertz emitter, known as the sensing plate. Silicon-on-sapphire (SOS) substrate, named as the sensing plate, was used to map the surface potential using the TCM system. The sensing plate (10 cm × 10 cm) is composed of a thin SiO_2_ layer, approximately a few nanometers thick, followed by a 500 nm Si layer, and a 500 μm Al_2_O_3_ substrate, as shown in Figure 1a. Figure 1b illustrates the optical configuration of the TCM setup. The system uses a femtosecond laser with a repetition rate of 80 MHz, a pulse width of 130 fs, and a central wavelength of 780 nm. As shown in Figure 2b, this laser beam is split into two paths: a pump beam and a probe beam. The pump beam, modulated at 2 kHz, is focused through a lens onto the sapphire side of the sensing plate to generate terahertz waves. These THz waves are then collected and directed by parabolic mirrors and focused on a super-hemispherical lens attached to the sensing element. Meanwhile, the probe beam is routed through a delay stage and directed into a detector to facilitate time-resolved measurements of the generated THz signals. The underlying measurement mechanism is as follows: due to doping and interface states at the Si/SiO_2_ boundary, a depletion layer electric field ($E_{d}$) is already established. When the femtosecond laser pulse excites the inside of the semiconductor of the sensing plate, carriers (electrons and/or holes) are generated and rapidly accelerated under the influence of $E_{d}$, resulting in a transient current. The time-varying current, in turn, emits electromagnetic radiation—terahertz waves—whose amplitude is proportional to the time derivative of the current. More detail on the THz generation process can be found in our previous studies [27,28,29,30,31]. Eventually, Equation (1) is derived, relating to the potential change, the depletion layer electric field, and the THz amplitude ($E_{THz}$):

(1) $$E_{THz}\propto \;E_{d}=\sqrt{\frac{2eN}{\varepsilon_{0}\varepsilon_{d}}\phi_{S}}$$

Key parameters in Equation (1) include e (the elementary charge), N (the charge density per unit area), $\varepsilon_{0}$ (the vacuum permittivity), $\varepsilon_{d}$ (the relative permittivity of the semiconductor), and $\phi_{S}$ (the surface potential). By measuring the THz amplitude, it is possible to detect changes in surface potential and, consequently, gain insights into the chemical interactions occurring at the surface.

### 2.3. Substrate Cleaning and Aptamer Modification

Prior to aptamer modification, the SOS substrate was sonicated in 99.5% acetone and 99.5% ethanol for 2 min each to remove surface contaminants. After sterilization, 200 mM of NaOH solution was added to the wells and shaken for 5 min to introduce hydroxyl groups on the SiO_2_ surface. Following the NaOH treatment, the wells were rinsed once with Milli-Q water. APTES (2% in Milli-Q) was then added, and the wells were shaken for 30 min to form an amine-functionalized surface. After removing the APTES solution and rinsing twice with Milli-Q water, BS3 (10 mM in PBS) was added and incubated at room temperature for 1 h to introduce reactive groups for aptamer immobilization. The BS3 solution was subsequently removed, and the wells were rinsed twice with Milli-Q water.

Serotonin aptamer (sequence: 5′-amnio link-CGACTGGTAGGCAGATAGGGG AAG CTG ATT CGA TGC GTG GGT CG-3′) and dopamine aptamer (sequence:5′-amnio link-CGACGCCAGTTTGAAGGTTCGTTCGCAGGTGTGGAGTGACGTCG), prepared at 1800 ppm in PBS or aCSF, were added to the wells and incubated for 6 h at room temperature, followed by 18 h at 4 °C. This procedure enabled the aptamers to covalently bind to the BS^3^-functionalized surface. The wells were then washed with PBS, and 0.1 M ethanolamine-HCl was added for 20 min at room temperature to block non-specific binding sites. After removal of the ethanolamine-HCl solution and washing with PBS, the sensing plates were ready for the neurotransmitter detection experiments. Figure 2a shows the modification process, and Figure 2b presents an AFM image of serotonin-binding DNA aptamers immobilized on an SOS substrate. The image reveals the nanoscale roughness and morphology of the functionalized surface, with the white dot structures representing the individual aptamers. The tertiary structures of DNA aptamers in PBS and aCSF were predicted using Alphafold 3 [32].

### 2.4. For Surface Potential Mapping and Real-Time Aptamer–Serotonin Interaction Monitoring

To evaluate the surface potential mapping of aptamer–serotonin/dopamine complexes, serotonin and dopamine solutions, varying from 100 ppt to 1000 ppb, were then sequentially introduced into the solution wells. After each addition, the samples were gently shaken and allowed to interact with the immobilized DNA aptamers for 20 min. Following incubation, the wells were rinsed with PBS, and a new THz amplitude distribution was measured.

To evaluate the real-time THz amplitude response across a range of serotonin concentrations, we first recorded a baseline THz intensity distribution prior to the analyte introduction. Serotonin solutions, varying from 1 ppb to 1000 ppb, respectively, were then introduced into the solution wells. Real-time THz amplitude changes were recorded.

In addition, we conducted a parallel series of measurements using artificial cerebrospinal fluid (aCSF) instead of PBS. By comparing the THz responses under these two conditions, we evaluated the influence of the divalent cations in aCSF on the sensitivity of serotonin and dopamine detection. This dual-environment approach provides valuable insights into how ionic composition can enhance or modulate the binding dynamics observed in THz amplitude maps.

### 2.5. 3D-Printed PDMS Microwell for Calcium Ion Measurements

To measure calcium ion concentration using TCM, a selective membrane that only reacts to calcium ions is prepared. The calcium ion-selective membrane is a membrane in which an ion-sensitive substance (ionophore) that selectively reacts with the target ion is dissolved in a suitable solvent and dispersed and retained in a base material softened by a plasticizer. In the calcium ion-selective membrane, polyvinyl chloride (PVC) serves as the base material, while dioctyl sebacate (DOS) acts as the plasticizer. Sodium ionophore II functions as the ionophore, and sodium tetrakis[3,5-bis(trifluoromethyl)phenyl]borate is used as the additive. The amounts of these materials are given in Table 1.

Since the density of DOS is 0.914 g/mL, 72 μL of DOS was mixed to contain 65.45 mg of DOS. The mixture was dissolved in THF (tetrahydrofuran; Wako Pure Chemical Industries, Ltd., Osaka, Japan). The calcium ion-selective membrane solution was prepared by mixing at 50 rpm for more than 24 h in a VMR-3R variable mixer (AS ONE Corporation, Osaka, Japan). The calcium ion-selective membrane solution was dropped on the SOS substrate one day before the measurement, and the solvent (THF) was evaporated and dried in the dark to form a membrane on the sensing plate. In this study, the wells are miniaturized with PDMS, and microwell-based measurements are performed. In TCM, the sensing plate is irradiated with a femtosecond laser pulse, and the potential change at the irradiated area is measured. The measurement area is therefore approximately the size of the laser focal spot (1 μm). Therefore, the measurement can be performed even if the well size is as small as the laser size. To measure calcium ion concentrations without a reference electrode, a calcium ion-sensitive membrane was applied to the sensing plate. PDMS wells (1 μL volume) were fabricated to minimize the sample volumes. Prior to the sample addition, a background THz amplitude was acquired. Then, 1 μL samples containing varying calcium ion concentrations (sodium ions: 1.4 × 10^−1^ mol/L; potassium ions: 4 × 10^−3^ mol/L) were introduced into the PDMS wells, and the THz amplitude was measured. The difference in THz amplitude before and after the sample addition was used to quantify the calcium ion concentration.

## 3. Results and Discussion

### 3.1. Mapping of the Surface Potential of DNA Aptamer–Serotonin Complexes

**Figure 3:**
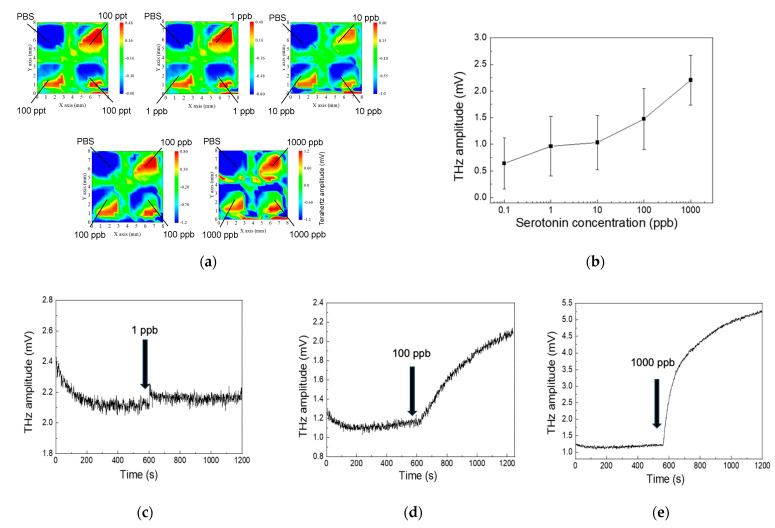
(**a**) Terahertz amplitude distribution at increasing serotonin concentrations; (**b**) concentration-dependent THz amplitude response to serotonin; (**c**) real-time THz response to 1 ppb serotonin; (**d**) real-time THz response to 100 ppb serotonin; (**e**) real-time THz response to 1000 ppb serotonin.

Figure 3a displays a series of two-dimensional THz amplitude maps obtained under varying conditions. Each map corresponds to a different serotonin concentration exposed to the DNA aptamer-functionalized SOS surface. The sequence includes a background measurement (i.e., phosphate-buffered saline, PBS) followed by serotonin solutions at 1 ppb, 10 ppb, 100 ppb, and 1000 ppb. The color scale indicates the THz amplitude, clearly illustrating how the THz response intensifies with increasing analyte concentrations. Figure 3c–e depict temporal response curves of the THz amplitude as the sensor is exposed to specific serotonin concentrations (1 ppb in (c), 100 ppb in (d), and 1000 ppb in (e)). Each graph shows the THz amplitude over time, with a vertical arrow indicating the point at which the target analyte is introduced. These time-series plots illustrate the speed and intensity of the DNA aptamer-modified SOS substrate’s reaction to increasing analyte concentrations, confirming the concentration-dependent behavior observed in earlier results.

### 3.2. Comparative Study of DNA Aptamer Conformational Changes and Terahertz Responses Changes in PBS and aCSF

In the comparative study of DNA aptamer conformational changes, the ionic composition of the surrounding medium plays a crucial role. Table 2 compares the primary ionic constituents of two commonly used physiological buffers: phosphate-buffered saline (PBS) and artificial cerebrospinal fluid (aCSF). While both solutions maintain phosphate-based buffering capacity, aCSF incorporates additional divalent ions such as Mg^2^^+^ and Ca^2^^+^. These ions can influence the folding, stability, and binding affinity of DNA aptamers. By examining aptamer behavior in PBS versus aCSF, the way ionic composition affects aptamer structure and function under conditions more closely resembling the in vivo environment can be better understood.

**Figure 4:**
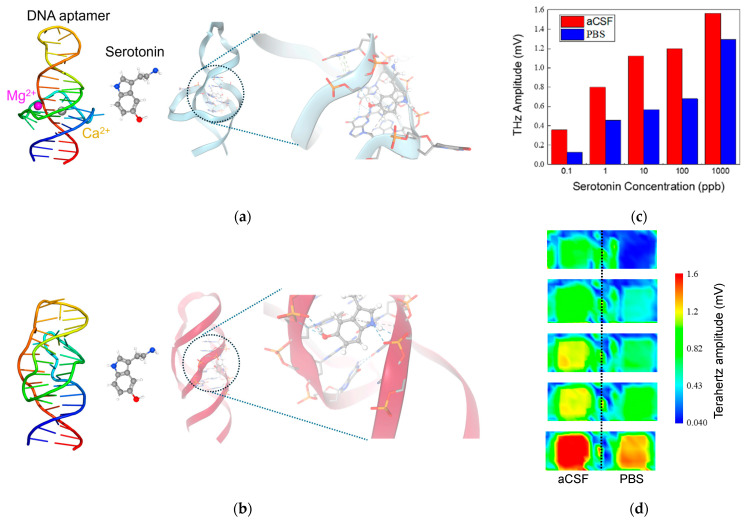
Molecular interactions and experimental analysis of DNA aptamer–serotonin complexes in aCSF and PBS. (**a**) Structural modeling in aCSF; (**b**) structural modeling in PBS; (**c**) experimental results in aCSF and PBS (over 200 data points measured on a single SOS substrate and averaged); (**d**) terahertz amplitude distribution mapping in aCSF and PBS.

Figure 4 integrates structural modeling, molecular interactions, and experimental sensing data to illustrate how the ionic environment influences serotonin binding to a DNA aptamer and the resulting THz amplitude response. In Figure 4a,b, molecular models generated via SwissDock simulations show the serotonin-binding DNA aptamer complexed with/without divalent cations (Mg^2^^+^ and Ca^2^^+^) [33,34]. Under artificial cerebrospinal fluid (aCSF) conditions, the aptamer–serotonin complex is stabilized by a combination of hydrogen bonds, hydrophobic interactions, and π-stacking interactions. In contrast, when the complex is formed in PBS, the primary stabilizing forces are hydrogen bonds and hydrophobic interactions, without significant π-stacking contributions. The 3D renderings highlight the aptamer’s tertiary structure and the precise binding orientation of serotonin within its binding pocket, illustrating how the presence of divalent cations in aCSF can promote more diverse and potentially stronger molecular interactions.

In Figure 4c, a comparative bar chart shows the THz amplitude responses recorded in aCSF (red bars) versus PBS (blue bars) at various serotonin concentrations. While the PBS measurements yield higher signals at lower concentrations, the aCSF environment produces enhanced responses at elevated serotonin levels. In Figure 4c, a series of THz amplitude distribution maps visualize the signal intensity upon different serotonin concentration introductions. The experimental results indicate that the THz amplitude obtained in aCSF is higher than in PBS, which aligns closely with the outcomes predicted by the modeling simulations.

**Figure 5:**
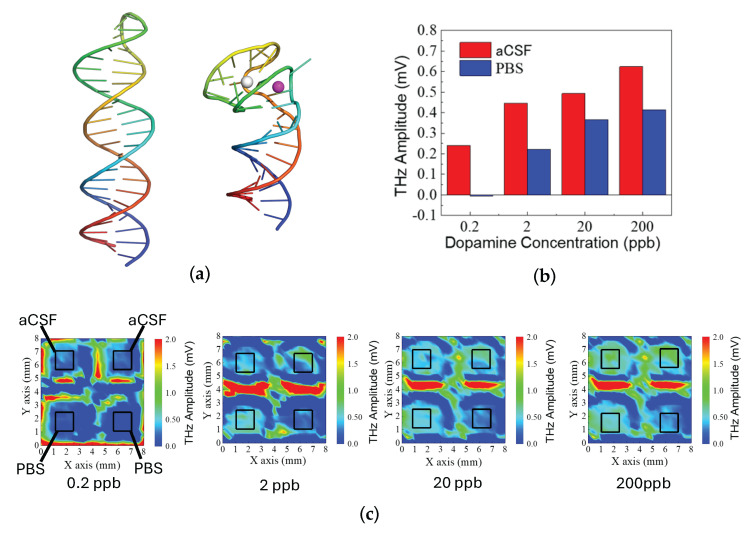
Molecular interactions and experimental analysis of DNA aptamer–dopamine complexes in aCSF and PBS. (**a**) Structural modeling in PBS and aCSF; (**b**) experimental results in aCSF and PBS (over 100 data points measured on a single SOS substrate and averaged); (**c**) terahertz amplitude distribution mapping in aCSF and PBS.

In Figure 5, consistent results were observed for the dopamine-binding DNA aptamer and its complex with dopamine. The predicted tertiary structures of the dopamine-binding DNA aptamer in PBS and aCSF are shown in Figure 5a, highlighting structural differences between the two environments. The terahertz measurement results, presented in Figure 5b, indicate that the terahertz amplitude was higher in aCSF compared to PBS, suggesting an enhanced interaction in the aCSF environment. Figure 5c displays terahertz amplitude distribution maps corresponding to dopamine concentrations of 20 ppb, 200 ppb, and 2000 ppb, illustrating the surface potential variations at different concentration levels.

### 3.3. PDMS Microwell-Based Membrane–Calcium Ion Interaction and Potential Mapping

Conventional ion detection methods, such as ion-selective electrodes (ISEs) and ion-sensitive field-effect transistors (ISFETs) [35,36,37], rely on reference electrodes, limiting their potential for miniaturization, integration, and reduced sample volumes. In contrast, TCM operates without the need for a reference electrode; therefore, it could emerge as a promising platform for accurate, non-invasive calcium ion concentration measurements, offering enhanced scalability and versatility over traditional ion measurement methods.

**Figure 6:**
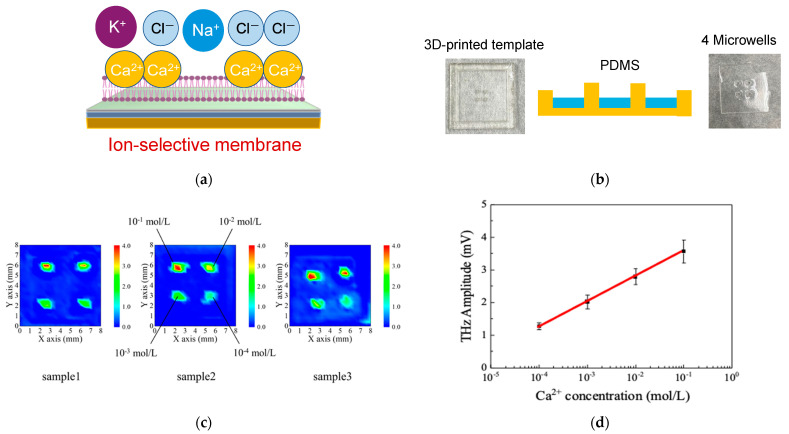
The results of calcium ion concentration measurements using PDMS microwells. (**a**) shows the differences before and after the calcium ion reaction. This confirms the change in terahertz wave intensity in the PDMS wells. (**b**) The mean value and standard deviation of each plot in (**b**), (**c**) presents THz intensity distribution maps for three different SOS substrates (samples 1, 2, and 3) exposed to varying Ca^2+^ concentrations (10^−1^ to 10^−^^4^ mol/L). The images depict how THz amplitude responds to increasing ion concentrations, demonstrating a highly linear correlation over a wide concentration range in (**d**).

Figure 6 demonstrates a terahertz-based approach for calcium ion detection using an SOS substrate coated with an ion-selective membrane and integrated with PDMS microwell structures. In Figure 6a, a schematic illustrates how the ion-selective membrane interacts with Ca^2^^+^ ions, altering the surface charge distribution and thus modulating the THz response. The presence of Ca^2^^+^ ions leads to a measurable change in THz wave intensity, enabling quantitative detection.

To create the microwell structures, PDMS was employed. As PDMS can be molded to replicate microscale features, 3D printing technology was used to form microwells with a volume of approximately 1 μL on the SOS substrate. These wells were then filled with the calcium ion-selective membrane solution (Figure 6b). The sample solutions contained calcium ions along with controlled concentrations of sodium (1.4 × 10^−1^ mol/L) and potassium (4 × 10^−3^) mol/L) ions. First, a background of the THz amplitude distribution map was acquired from the PDMS wells before the reaction. Next, the calcium-containing sample solution (1 μL) was introduced, and a second THz amplitude distribution was obtained. By subtracting the background map, the changes attributable to calcium ions alone could be identified. Figure 6c presents THz intensity distribution maps for three different SOS substrates (samples 1, 2, and 3) exposed to varying Ca^2^^+^ concentrations (10^−^^1^ to 10^−^^4^ mol/L). The images depict how THz amplitude responds to increasing ion concentrations, demonstrating a highly linear correlation over a wide concentration range in Figure 6d. The observed increase in THz amplitude with rising calcium concentration confirms that even microvolume solutions (1 μL) can be accurately assessed.

By integrating an ion-selective membrane onto an SOS substrate and PDMS microwells, we achieved sensitive and linear detection of calcium ions by monitoring surface potential changes through THz measurements. This approach underscores the potential of THz sensing platforms for rapid, small-scale ion monitoring.

## 4. Conclusions

This study demonstrated the feasibility and effectiveness of TCM in mapping surface potential changes resulting from DNA aptamer–neurochemical and membrane–ion interactions on a silicon-on-sapphire (SOS) substrate. By leveraging femtosecond laser pulses to generate terahertz waves and correlating their amplitude with underlying surface potential, we achieved label-free, sensitive detection of molecular and ionic species in microvolume samples.

The results reveal that neurochemicals like serotonin and dopamine can be reliably monitored through TCM by measuring the terahertz amplitude. DNA aptamers proved to be promising elements, enabling sensitive detection across a broad concentration range. Additionally, the inclusion of artificial cerebrospinal fluid (aCSF) in the measurement environment highlighted the role of divalent cations, such as Mg^2^^+^ and Ca^2^^+^, in enhancing detection sensitivity. These observations underscore the critical influence of ionic composition on sensor performance. Results on calcium ion detection demonstrated that integrating ion-selective membranes within PDMS microwells enables precise and linear correlations between terahertz amplitude and ion concentrations, further showing the versatility of TCM for analyzing diverse analyte classes.

In summary, our findings highlight TCM’s potential as a powerful diagnostic and analytical tool. By enabling direct visualization and quantification of dynamic biochemical processes, TCM is well-positioned to advance surface-potential-driven research in biomedical applications, as well as in materials science and related fields.

## Figures and Tables

**Table 1:** Composition of calcium ion selective membrane.

Polyvinyl chloride (Base Polymer)	33 wt%
Bis(2-ethylhexyl) sebacate (Plasticizer)	65.5 wt%
N,N,N′,N′-Tetra(cyclohexyl)diglycolic acid diamide (Ionophore)	1 wt%
Sodium tetrakis[3,5-bis(trifluoromethyl)phenyl] borate (Additive)	0.5 wt%
Tetrahydrofuran (Solvent)	588 μL

**Table 2:** Composition of phosphate-buffered saline (PBS) and artificial cerebrospinal fluid (aCSF).

	PBS	aCSF
**Composition**	**Concentration (mM)**	
NaCl	137	147
KCl	2.7	3.5
Na_2_HPO_4_	10	10
KH_2_PO_4_	1.8	2.5
MgCl_2_	-	1.2
CaCl_2_	-	1.0

## Data Availability

Data supporting the findings of this study are available from the corresponding author, J.W., upon reasonable request.
